# Lipid Profile Changes During the Development of *Artemia franciscana*, From Cysts to the First Two Naupliar Stages

**DOI:** 10.3389/fphys.2018.01872

**Published:** 2019-01-22

**Authors:** Patrizia Lopalco, Simona Lobasso, Ruy Miguel Alfama Lopes-dos-Santos, Gilbert Van Stappen, Angela Corcelli

**Affiliations:** ^1^Department of Basic Medical Sciences, Neuroscience and Sense Organs, University of Bari Aldo Moro, Bari, Italy; ^2^Laboratory of Aquaculture & Artemia Reference Center, Ghent University, Ghent, Belgium

**Keywords:** *Artemia franciscana*, phospholipids, lysophospholipids, neutral lipids, MALDI-TOF/MS, TLC

## Abstract

The brine shrimp *Artemia* is an interesting experimental system for studies of developmental processes. Hatching of dormant cysts gives rise to shrimp larvae called nauplii, characterized by numerous naupliar stages representing the first forms of brine shrimp life cycle. Here combined Thin Layer Chromatography (TLC) and Matrix-Assisted Laser Desorption Ionization-Time-of-Flight/Mass Spectrometry (MALDI-TOF/MS) analyses have been performed to gain information on the lipid profiles of cysts and two naupliar stages. Lipid bands isolated after preparative TLC of the lipid extracts have been analyzed to detect various species of each lipid class; in addition Post*-*Source Decay (PSD) analyses allowed the identification of phospholipid chains. We compared the relative abundance of various polar and neutral lipid species in the lipid extracts, proving for the first time that during the development of nauplii there is an increase of cardiolipin (CL) and lysophospholipid levels; in parallel, the amount of phosphatidylcholine (PC) decreases. In addition, as regards neutral lipids, we found an increase of diacylglycerols (DAGs) in correspondence of the decrease of triacylglycerols (TAGs). Data reflect the fact that naupliar stages, being an active form of life, are more metabolically active and offer a platform to develop further studies on the importance of lipid metabolic pathways and bioactive lipids during the development.

## Introduction

*Artemia franciscana* is a planktonic crustacean inhabiting natural salt lakes and salterns. Their larvae (nauplii) represent the most commonly used live food in aquaculture (Fisheries and Aquaculture Resources Use and Conservation Division, 2017). Apart from practical applications, the brine shrimps of the genus *Artemia* are interesting organisms from the physiological point of view. The different stages of the life cycle of *Artemia* offer many examples of remarkable physiological mechanisms.

For example, the state of anaerobic quiescence in *A. franciscana* embryos represents the case of the most profound metabolic arrest reported in invertebrates. The mechanism of quiescence of encysted embryos is unique involving acidification of the intracellular milieu as major factor controlling catabolic and anabolic down regulation; recovery from the metabolic arrest requires re-sequestration of the protons with a vacuolar-type ATPase (V-ATPase) (Hand et al., 2011). Cysts remain viable for years and produce nauplii within 24 h after hydration adopting fast metabolic changes to sustain the transformation of tissues.

During the differentiation of *Artemia* cysts, morphological and functional changes of mitochondria occur. Immediately after the hydration of cysts, at the beginning of the differentiation, significant changes in the mitochondrial respiratory capacity and marked changes in the morphology of the inner membrane have been observed (Schmitt et al., 1973).

*Artemia* nauplii are capable of regulating the ionic composition of the hemolymph against wide ranges of salinities thanks to specialized epithelia. It is well known that brine shrimps have the ability to adapt and survive under extreme salinities (up to 15% salinity or 2M salts) as well as in 10% seawater. In hyperosmotic media the gut absorbs ions and water and gills secrete ions; in fresh water, gills absorb ions and water in excess is eliminated with urine.

Furthermore because of common environment, the association of extreme halophilic microorganisms, such as halophilic Archaea, with *Artemia* is considered of ecological and physiological interest, given that specific microbes may be important in the brine shrimp life cycle and the surrounding food web (Riddle et al., 2013; Rahmani et al., 2016).

Recently, the lipidome of cysts of *A. franciscana* has been studied in parallel with lipids of mitochondria isolated from cysts by high resolution shotgun lipidomics, aiming to gain basic knowledge in the elucidation of actionable extremophilia-affording proteins, such as on the late embryogenesis abundant proteins (LEA), which are known to interact with lipid membranes (Chen et al., 2016). LEA proteins are particularly protective of mitochondrial membranes against dehydration damage (Tolleter et al., 2010).

The study of Chen et al. (2016) differentiates itself from previous lipid literature about *Artemia;* in effect, it represents the first lipidomic study on the *Artemia* eggs offering detailed information on lipid classes, on diversity within the lipid classes and on lipid proportions. Indeed, most of the previous analytical reports on lipids of *Artemia* are mainly related to nutritional analyses in aquaculture, given that the species is used as instant live food (Abatzopoulos et al., 2002). Therefore, fatty acid composition of *Artemia* has been frequently studied in the past because its determination is important to assess the nutritional quality of a source of *Artemia*.

As regards nutritional issues, it is known that *Artemia* sp. naturally possesses high contents of neutral lipids and low content of long-chain polyunsaturated fatty acids (LC-PUFA), such as 20:5n-3 (EPA), and especially 22:6n-3 (DHA), which are essential fatty acids for normal development of marine fish larvae (Sargent et al., 1999). In this respect, enrichment with fatty acids of *Artemia* sp. was used to tailor its lipid composition toward the nutritional needs of marine larvae (Webster and Lovell, 1990; Van Stappen, 1996; Sargent et al., 1999; Reis et al., 2017).

Specific lipid classes can also be important in fish and crustacean nutrition. In spite of this, until few years ago, information on the lipid class composition of *Artemia* was relatively scarce.

The present study reports novel data and information on lipids of *Artemia* by comparing the lipid composition of three different forms of life: cysts (C) and two naupliar stages, namely nauplii instar I (NI) and nauplii instar II (NII).

Here we have used a semiquantitative approach in the lipid analyses by combining TLC analyses and MALDI-TOF/MS. Our analytical approach is suited to give information on various species in different classes of polar and neutral lipids and on the proportion of different lipids in the three different forms of life of *Artemia*.

The study of the modification of the lipid composition during the development represents the basis to evaluate the changes in lipid metabolic pathways and, more generally, to understand the contribution of lipids to the development processes.

## Materials and Methods

### Materials

The matrix used for MALDI-TOF/MS analyses was 9-Aminoacridine hemihydrate (9-AA) and was purchased from Acros Organics (Morris Plains, NJ, United States). All organic solvents used in extraction and MS analyses were commercially distilled, of the highest available purity, and purchased from Sigma-Aldrich, J. T. Baker, or Carlo Erba. The following commercial glycerophospholipids (used as standards): 1,2-dimyristoyl-*sn*-glycero-3-phosphate, 1,2-dimyristoyl-*sn*-glycero-3-phospho-(1′-rac-glycerol), 1,2-dimyristoyl-*sn*-glycero-3-phospho-L-serine, 1,2-diphytanoyl-*sn*-glycero-3-phosphoethanolamine, 1′,3′-bis[1,2-dimyristoyl-*sn*-glycero-3-phospho]-*sn*-glycerol, 1′,3′-bis[1,2-dioleoyl-*sn*-glycero-3-phospho]-*sn*-glycerol were purchased from Avanti Polar Lipids, Inc. (Alabaster, AL, United States).

### Preparation of *Artemia* Decapsulated Cysts and Nauplii

*Artemia* decapsulated cysts and nauplii samples were obtained according to the protocol as described by Sorgeloos et al. (1977). Briefly, *Artemia franciscana* cysts, originating from the Great Salt Lake, Utah, United States (Ocean Nutrition, batch L19516012), were hydrated in sterile distilled water for 1.5 h. Decapsulated cysts were obtained using 32% NaOH and 50% NaOCl. The reaction was stopped after 2–4 min by adding 1% Na_2_S_2_O_3_. Decapsulated cysts were then washed with distilled water, being part sampled and frozen for analysis and the rest resuspended in an aerated glass cone containing filtered (0.22 μm) and autoclaved seawater, and then incubated at 28°C with constant illumination (approximately 2000 lux) for hatching. After 22 and 30 h of incubation, respectively, the instar I and instar II were harvested on a sieve, washed with distilled water and frozen for analysis.

### Lipid Extraction

*Artemia* decapsulated cysts and nauplii samples were thawed and homogenized 20-fold on ice with a Potter homogenizer; then total lipids were extracted using the Bligh and Dyer method (Bligh and Dyer, 1959). The extracts were dried under N_2_ before weighing and then dissolved in chloroform (final concentration 10 mg/ml). Fractionation of the total lipid extract by the procedure of cold acetone precipitation yielded a fraction enriched in neutral lipids (Kates, 1986).

### TLC Analyses

Total lipid extracts were analyzed by thin layer chromatography (TLC) on silica gel 60A plates (Merck, 20 × 10 cm, layer thickness 0.2 mm). The plates were washed twice with chloroform/methanol (1:1, by volume) and activated at 180°C before use. Polar lipids were eluted with Solvent A (chloroform/methanol/acetic acid/water 85:15:10:3.5, by volume); the neutral lipids were separated by TLC in Solvent B (hexane/diethyl ether/acetic acid, 70:30:1, by volume).

Lipid detection was carried out by spraying the plate with 5% sulfuric acid in water, followed by charring at 180*°*C for 5 min, or exposing the TLC plate to iodine vapor, for staining all classes of lipids. Moreover, the following stainings were performed in order to identify the different lipid classes present in the TLC bands: (i) molybdenum blue spray reagent (Sigma-Aldrich) specific for phospholipids, and (ii) ninhydrin solution, prepared dissolving 0.25 g of reagent grade ninhydrin in 100 ml of acetone-lutidine (9:1, by volume), for phosphatides or lipids carrying a free amino group (Kates, 1986).

The estimation of the content of individual polar and neutral lipids of the total lipid extracts was performed by video densitometry analysis of spots on TLC, obtained after averaging three replicates of C, NI, and NII (*ImageJ software)*. For quantitative determination of lysophosphatidylethanolamine (LPE) content, a standard curve was used. The standard curve of LPE authentic standard stained with ninhydrin solution was linear in the concentration range 0.5–5.0 μg. In addition, in this case the staining intensity was evaluated by densitometry using ImageJ software.

### Statistical Analysis

All densitometric values are expressed as mean ± standard deviation (SD). One-way ANOVA was used to compare results of data from the three conditions (C, NI, and NII) followed by Tukey’s HSD *post hoc* test. *P*-values less than 0.05 were considered significant. Statistical analysis was made in R environment (R Core Team, 2012).

### Isolation and Purification of Individual Lipids From the Total Extract

In order to analyze in detail the various components of the lipid extracts, bands present on plates were scraped and lipids extracted from silica were then analyzed by positive and negative ion modes MS.

Briefly the polar lipid components of the total lipid extracts of NII were separated by TLC (Merck 20 × 10 cm × 0.2 mm thick layer, glass plates) in Solvent A. While, the neutral lipids components were separated by TLC in solvent B. Lipids were visualized by staining with iodine vapor and were eluted and recovered from the scraped silica, as previously described (Lobasso et al., 2012; Lopalco et al., 2017). Isolated and purified phospholipids were dissolved in chloroform (1 mg/ml).

### Preparation of Lipid Extracts for MALDI-TOF/MS Lipid Analyses

Three microliter of lipid samples in chloroform solution were diluted in 27 μl of 2-propanol/acetonitrile (60/40, by volume), then 10 μl of the diluted solution were mixed with 10 μl of matrix solution (9-AA, 10 mg/ml in 2-propanol/acetonitrile 60/40, by volume), as previously described (Sun et al., 2008; Angelini et al., 2015). At variance, spectra of neutral lipids were acquired by preparing the same matrix solution in the presence of sodium acetate, as previously described (Sun et al., 2008).

The resulting lipids-matrix solution was then spotted onto the MALDI target (Micro Scout Plate, MSP 96 ground steel target) in droplets of 0.35 μl and analyzed as described below.

After the evaporation of the matrix solvent, the samples are ready to be directly analyzed with MALDI-TOF/MS.

### MALDI-TOF Mass Spectrometry

MALDI-TOF mass spectra of lipid extracts were generally acquired on a Bruker Microflex LRF mass spectrometer (Bruker Daltonics, Bremen, Germany). The system utilizes a pulsed nitrogen laser, emitting at 337 nm, the extraction voltage was 20 kV and gated matrix suppression was applied to prevent detector saturation. For each mass spectrum, 2000 single laser shots (sum of 4 × 500) were averaged. The laser fluence was kept about 5% above threshold to have a good signal-to-noise ratio. All spectra were acquired in reflector mode (detection range: 200–2000 mass/charge, *m/z*) using the delayed pulsed extraction; spectra were acquired in negative and positive ion modes. Spectral mass resolutions and signal-to-noise ratios were determined by the software for the instrument: “Flex Analysis 3.3” (Bruker Daltonics).

A mix containing 1,2-dimyristoyl-*sn*-glycero-3-phosphate, 1,2-dimyristoyl-*sn*-glycero-3-phospho-(1′-rac-glycerol), 1,2-dimyristoyl-*sn*-glycero-3-phospho-L-serine, 1,2-diphytanoyl-*sn*-glycero-3-phosphoethanolamine, 1′,3′-bis[1,2-dimyristoyl-*sn*-glycero-3-phospho]-*sn*-glycerol, 1′,3′-bis[1,2-dioleoyl-*sn*-glycero-3-phospho]-*sn*-glycerol was always spotted next to the sample as external standard and an external calibration was performed before each measurement in negative ion mode; the mass range of the authentic standards is 590–1450 atomic mass units *(amu)*. A mix containing 1,2-distearoyl-sn-glycero-3-phosphocholine, 1,2-dimyristoleoyl-sn-glycero-3-phosphocholine, 1,2-di-O-phytanyl-sn-glycero-3-phosphocholine was always spotted next to the sample as external standard and an external calibration was performed before each measurement in positive ion mode.

Post-Source Decay (PSD) spectra were acquired on a Bruker Microflex mass spectrometer (Bruker Daltonics, Bremen, Germany), as previously described (Fuchs et al., 2007). Briefly, the precursor ions were isolated using a time ion selector. The fragment ions were refocused onto the detector by stepping the voltage applied to the reflectron in appropriate increments. This was done automatically by using the “FAST” (fragment analysis and structural TOF) subroutine of the Flex Analysis software. Mass accuracy of our instrument is 200 ppm (external calibration). A specific lipid database (Lipid Maps Database)^1^ (LIPID MAPS Lipidomics Gateway, 2018) was used to facilitate and confirm the assignment of lipid species.

## Results

### Thin Layer Chromatography Analyses Reveal That Lysophospholipids and Cardiolipin Increase in Nauplii

**FIGURE 1:**
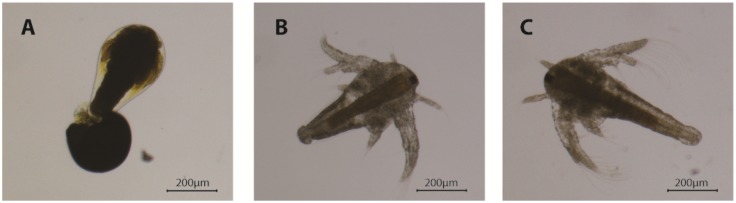
Photos of three stages of *Artemia franciscana* during the development: **(A)** *Artemia* in the umbrella stage hatching from cyst, **(B)** *Artemia* nauplii Instar I, **(C)** *Artemia* nauplii Instar II.

In order to gain information on lipid profiles during the development of *A. franciscana* (Figure 1), we analyzed the total lipid extracts of cysts and two naupliar stages by TLC.

**FIGURE 2:**
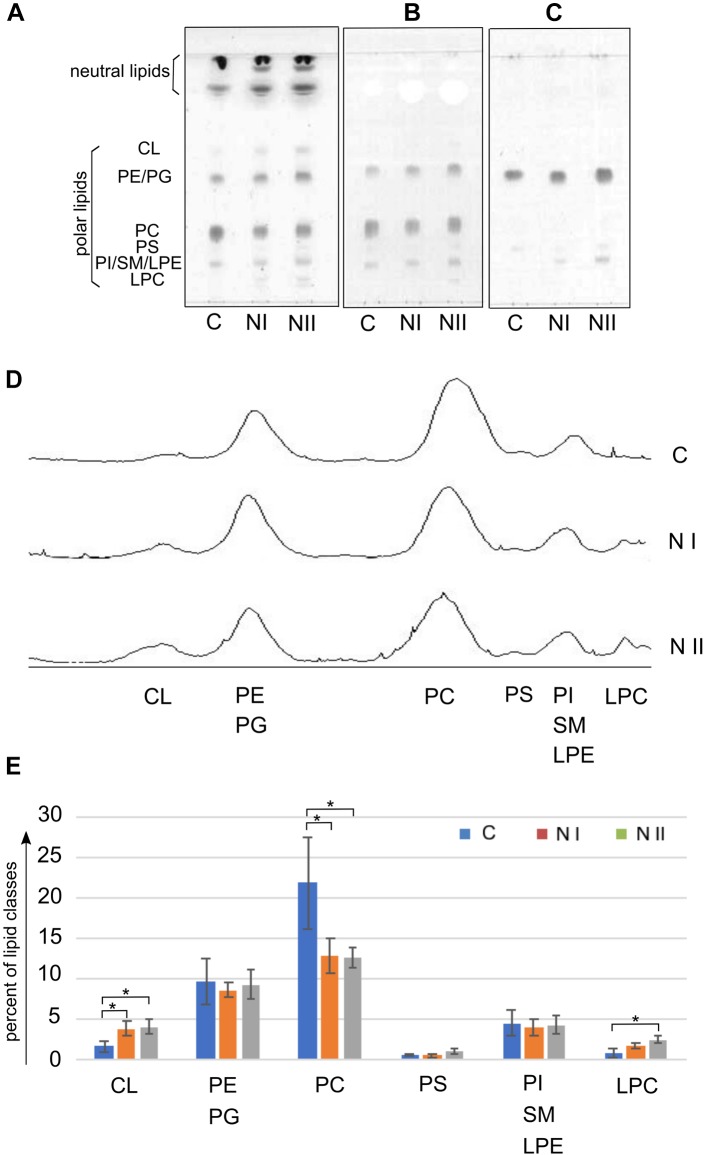
Polar lipid classes in *Artemia franciscana*: **(A–C)** show TLC profiles of the total lipid extracts of Cysts (C), nauplii instar I (NI) and nauplii instar II (NII); 80 μg of total lipid extract was loaded in each lane. Lipids were stained by: **(A)** 5% sulfuric acid in water, followed by charring at 180°C for 5 min, for all classes of lipids; **(B)** molybdenum blue spray reagent, for phospholipids; **(C)** ninhydrin solution, 0.25% in acetone-lutidine (9:1, by volume), for phosphatides or lipids having a free amino group. Lipid bands are indicated by their abbreviations: cardiolipin (CL), phosphatidylethanolamine (PE), phosphatidylglycerol (PG), phosphatidylcholine (PC), phosphatidylserine (PS), phosphatidylinositol (PI), sphingomyelin (SM), lysophosphatidylethanolamine (LPE), and lysophosphatidylcholine (LPC). Large overlapping bands of neutral lipids are present close to the front. **(D)** Shows densitometric evaluations of the three TLC profiles in **(A).** **(E)** Illustrates the relative densitometric intensity of CL, PE/PG, PC, PS, PI/SM/LPE and LPC bands after averaging the peak intensities in the chromatograms of three replicates of C, NI, and NII; values on the y axis (in percent) were estimated by video densitometry, as described in the Materials and Methods section. ^∗^*P* < 0.05 byTukey’s *post hoc* test.

Figure 2 illustrates the polar lipid profiles of cysts (C), nauplii instar I (NI), and nauplii instar II (NII). In Figures 2A–C we show the TLC lipid profiles stained with different reagents specific for: all classes of lipids, phospholipids and phosphatides or lipids carrying a free amino group, respectively.

In the first TLC plate (Figure 2A on the left) the lipid bands were identified by their retention factor (R_f_) values relative to authentic standards (not shown). The other specific stainings for phospholipids and free amino group containing lipids helped us to recognize minor lipids in the bands of higher retention factors.

By examining the plate on the left, it can be seen that, besides the bands of polar lipids, large overlapping bands of neutral lipids are present close to the front (see section TLC of Neutral Lipids: Free Fatty Acids and DAGs Are More Abundant in the Nauplii Than in the Cysts).

The polar lipid bands were assigned (in R_f_ order from the top) to: cardiolipin (CL), phosphatidylethanolamine (PE) comigrating with phosphatidylglycerol (PG), phosphatidylcholine (PC), phosphatidylserine (PS), phosphatidylinositol (PI) almost comigrating with lysophosphatidylethanolamine (LPE) and sphingomyelin (SM), and lysophosphatidylcholine (LPC). The comparison of the TLC lipid profiles of three different stages of *A. franciscana* suggests that the lipid composition of naupliar stages is different from that of cysts. CL and lysophospholipids, such as LPC (first band from the bottom in Figures 2A,B) and LPE (first band from the bottom in Figure 2C) are minor lipid components of C and increase during the development from C to NII. Changes in LPE are clearly visible and distinguishable in the plate on the right stained with ninhydrin (Figure 2C). We could also identify PS as a pale band close to LPE and PE in the middle of the TLC plate shown in Figure 2C.

In the Figures 2D,E video densitometric analyses of polar lipids are reported. The peaks of the three chromatograms in the Figure 2D correspond to the relative intensity of polar lipid bands separated by TLC (plate in Figure 2A).

The histogram in Figure 2E shows the relative densitometric intensity (as percent) of CL, PE/PG, PC, PS, PI/SM/LPE, and LPC bands after averaging the chromatograms of three replicates of C, NI, and NII profiles. Percent values of each polar lipids together with results of statistical analysis are reported in the Table 1. The most important changes in the proportion of polar lipids during the development are the following: CL and LPC increase significantly from C to NII; PC is more abundant in the cysts and decrease in the two naupliar stages (from about 22% in the cysts to about 13% in the naupliar stages); PE plus PG corresponds to about 9–10% of total lipids in all three developing stages. No significant differences in the low PS content of all the samples have been found. These data indicate that total polar lipids correspond to 30–40%, while the neutral lipids represent the preponderant amount (almost 60–70%).

**Table 1:** Polar lipid classes identified in cysts, nauplii I, and nauplii II.

Lipid classes	% total lipid		

	Cysts	Nauplii I	Nauplii II
LPC	0.9 ± 0.6	1.8 ± 0.4	2.6 ± 0.5
PI + SM + LPE	4.6 ± 1.5	4.0 ± 1.0	4.3 ± 1.1
PS	0.8 ± 0.1	0.6 ± 0.2	1.0 ± 0.3
PC	21.8 ± 5.6	12.9 ± 2.2	12.7 ± 1.3
PE + PG	9.7 ± 2.8	8.6 ± 0.9	9.3 ± 1.3
CL	1.8 ± 0.7	3.9 ± 0.8	4.1 ± 0.9


*Data are means ± SD of triplicates expressed in percent total lipids. One-way ANOVA [F_(2,__6)_= 8.35, p = 0.02 for LPC; F_(2,__6)_= 6.42, p = 0.03 for PC; F_(2,6)_ = 7.76, p = 0.02 for CL] was performed followed by post hoc Tukey’s test.*

To evaluate the amount of LPE, we considered the plate stained with ninhydrin (Figure 2C) as the overlapping PI and SM bands do not stain. LPE is absent in C, while it amounts to 0.8 ± 4.2 μg and 2.3 ± 1.4 μg in 100 micrograms of total lipids in NI and NII, respectively, as estimated by interpolation with a standard curve (not shown, details in Materials and Methods section).

Noteworthy an higher content of the mitochondrial lipid marker CL (clearly seen in the data reported in Figures 2A,D,E) was observed in both the lipid profiles of the two naupliar stages, suggesting an increase in demanding of metabolic energy during the development from cysts to naupliar stages.

### MALDI-TOF/MS Analyses of Individual Bands Isolated From the Total Lipid Extract of Nauplii Instar II by Preparative TLC

Total lipid extracts of cysts, nauplii instar I and nauplii instar II were analyzed by MALDI-TOF/MS in negative and positive ion mode (not shown). In our experimental conditions, the mass spectra of the total lipid extracts had limited reproducibility, likely due to the preponderant presence of neutral lipids as shown before.

The lipid mass spectrometry analyses after chromatographic separation offer the opportunity to identify minor lipids such as cardiolipins, plasmalogens, and lysophospholipids whose signals were barely distinguishable from the noise in the MALDI-TOF/MS lipid profiles of the total lipid extracts (not shown).

**FIGURE 3:**
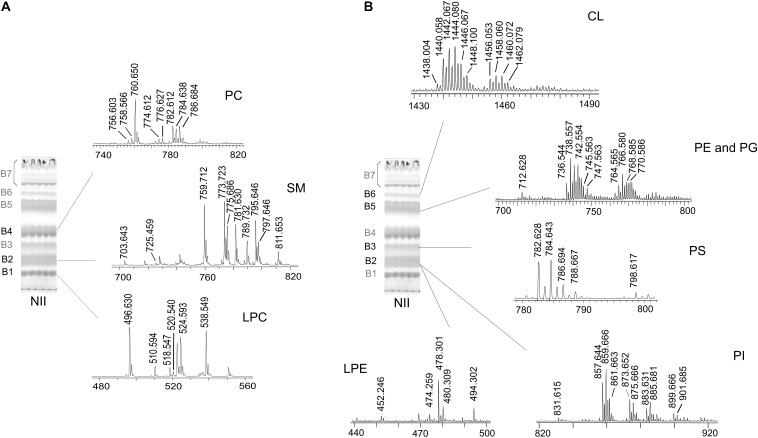
MALDI-TOF/MS analyses of individual lipid bands isolated from nauplii instar II by TLC. The total lipid extract of NII was loaded on the plate (160 μg per each lane). TLC was stained with iodine vapors (temporary staining of all classes of lipids). The same chromatography plate is shown on the left of both panels; the R_f_ value of each band corresponds to the R_f_ value of TLC bands in Figure 2A. Seven lipid bands (B1–B7) were marked with a pencil and silica was scraped; lipid bands were extracted from silica and analyzed by MALDI-TOF/MS. **(A)** Shows the MALDI/TOF-MS spectra of the lipid bands acquired in positive ion mode; B1, B2, and B4 corresponds to: lysophosphatidylcholine (LPC), sphingomyelin (SM), and phosphatidylcholine (PC), respectively. **(B)** Shows the MALDI/TOF-MS spectra of the lipid bands acquired in negative ion mode; B2 corresponds to lysophosphatidylethanolamine (LPE) and phosphatidylinositol (PI), B3 to phosphatidylserine (PS), B5 to phosphatidylethanolamine (PE) and phosphatidylglycerol (PG) and B6 to cardiolipin (CL). The detailed list of detected peaks is shown in Tables 2, 3.

To further support the TLC assignments and gain detailed information on lipid species, the various lipid bands were isolated by preparative TLC (see plate on the left of Figures 3A,B) and then analyzed by MALDI-TOF/MS (mass spectra of bands are on the right of the plate in Figures 3A,B). The separation of bands refers to the total lipid extract of nauplii instar II, where CL and lysocompounds are more abundant.

Seven bands (B1–B7) of polar lipids stained with iodine vapors were scraped from the plate; then lipids were extracted from silica (Bligh and Dyer, 1959). TLC bands were analyzed by MALDI-TOF/MS in positive and negative ion mode, confirming the presence of the glycerophospholipids previously identified in the total lipid extracts by TLC (Figure 2).

Figure 3A shows the MALDI-TOF mass spectra of lipid bands B1, B2, and B4, in positive ion mode; MALDI-TOF mass spectra acquired in negative ion mode refer to the lipid bands B2, B3, B5, and B6 in Figure 3B. A detailed list of all peaks present in mass spectra acquired in positive and negative ion mode, corresponding to the lipid species present in the lipid bands and/or in the total lipid extracts, is reported in Tables 2, 3, respectively.

**Table 2:** Assignments of *m/z* values in MALDI–TOF mass spectra detected in positive ion mode of lipid bands isolated from nauplii instar II.

Lipid classes	*m/z* value	[M+H]^+^	Assignment
			
LPC	496.630	496.340	16:0
	520.540	520.340	18:2
	524.593	524.371	18:0
LPC (+Na^+^)	518.547	518.322	16:0
Plasmenyl-LPC	510.594	510.392	18:0
	538.549	538.423	20:0
PC	756.603	756.554	34:3
	758.625	758.569	34:2
	760.650	760.585	34:1
	782.597	782.569	36:4
	784.638	784.585	36:3
	786.448	786.601	36:2
PC (+Na^+^)	782.597	782.567	34:1
Plasmenyl-PC	774.612	774.637	36:1
	776.627	776.653	36:0
SM	703.643	703.575	d16:1–18:0
	759.712	759.637	d16:1–22:0
	773.723	773.653	d17:1–22:0
	775.701	775.669	d17:0–22:0
	789.732	789.684	d18:0–22:0
	811.657	811.669	d20:2–22:1
SM (+ Na^+^)	725.500	725.557	d16:1–18:0
	781.630	781.619	d16:1–22:0
	795.646	795.635	d17:1–22:0
	797.646	797.651	d17:0–22:0
TAG (+Na^+^)	828.461	827.720	48:1
	830.466	829.736	48:0
	877.639	877.736	52:4
	901.653	901.736	54:6
	903.707	903.752	54:5
	911.675	911.815	54:1
	913.542	913.830	54:0
DAG (+Na^+^)	587.367	587.475	32:2
	615.304	615.507	34:2
	639.313	639.507	36:4
	641.279	641.522	36:3


*The numbers (x:y) denote the total length (as carbon numbers) and number of double bonds of acyl chains, respectively.*

**Table 3:** Assignments of *m/z* values in MALDI –TOF mass spectra detected in negative ion mode of lipid bands isolated from nauplii instar II.

Lipid classes	*m/z* value	[M-H]^-^	Assignment
			
LPE	452.246	452.278	16:0
	474.268	474.262	18:3
	478.298	478.294	18:1
	480.309	480.310	18:0
Plasmenyl-LPE	494.302	494.362	20:0
PI	831.612	831.503	34:3
	857.644	857.519	36:4
	859.666	859.534	36:3
	861.676	861.550	36:2
	883.675	883.534	38:5
	885.681	885.550	38:4
	889.659	889.581	38:2
Plasmenyl-PI	873.652	873.586	38:3
	875.667	875.602	38:2
	877.673	877.617	38:1
	899.666	899.602	40:4
	901.685	901.617	40:3
PS	780.609	780.482	36:5
	782.628	782.498	36:4
	784.643	784.513	36:3
	786.649	786.529	36:2
	788.667	788.545	36:1
PE	712.516	712.492	34:3
	736.544	736.544	36:5
	738.557	738.508	36:4
	740.565	740.524	36:3
	742.582	742.540	36:2
	762.546	762.508	38:6
	764.565	764.524	38:5
	766.580	766.540	38:4
	768.585	768.555	38:3
	770.586	770.570	38:2
PG	745.563	745.502	34:2
	747.563	747.518	34:1
CL	1438.033	1437.889	72:13
	1440.056	1439.902	72:12
	1442.067	1441.918	72:11
	1444.080	1443.934	72:10
	1446.091	1445.949	72:9
	1448.100	1447.965	72:8
	1456.053	1456.028	72:4
	1458.053	1458.043	72:3
	1460.072	1460.059	72:2
	1462.079	1462.074	72:1


*The numbers (x:y) denote the total length (as carbon numbers) and number of double bonds of acyl chains, respectively.*

LPC was recognized in the band of highest retention factor on TLC (B1) (Figure 3A). Positive ion mode MALDI-TOF/MS analyses of B1 revealed peaks at *m/z* 496.6, 520.5, and 524.5, corresponding to LPC in their protonated form (16:0, 18:2, 18:0, respectively). Furthermore, the peak at *m/z* 518.5 corresponds to the sodiated form of LPC (16:0) and finally the peaks at *m/z* 510.5 and 538.5 correspond to the plasmenyl-LPC (18:0 and 20:0, respectively).

The mass spectrum of B2, in positive ion mode, revealed the main peaks at *m/z* 759.7, 773.7, 775.7, 789.7, and 811.6 corresponding to the protonated form of SM species and minor peaks at *m/z* 781.6, 795.7, and 797.6 corresponding to the sodiated form of other SM species (listed in Table 2). These data are an agreement of those obtained by Chen et al. (2016) and Kojima et al. (2010).

The mass spectrum of B4, acquired in positive ion mode, shows main peaks at *m/z* 760.6, 782.6, 784.6, and 786.7, corresponding to protonated form of PC species with different chain length (34:1, 36:4, 36:3, and 36:2, respectively); the peak at *m/z* 782.6 may also correspond to the sodiated form of PC (34:1); minor peaks at *m/z* 774.6 and 776.6 are attributable to plasmenyl-PC (36:1 and 36:0, respectively).

B7 contains neutral lipids corresponding to about 60% of total lipid content, as previously shown; more details about these lipids will be given in the next section TLC of Neutral Lipids: Free Fatty Acids and DAGs Are More Abundant in the Nauplii Than in the Cysts (see Figure 5).

B2 analyzed in negative ion mode shows peaks attributable to both PI and LPE (Figure 3B). In the mass spectrum corresponding to PI, the main peaks are at *m/z* 857.6, 859.7, and 885.7 referable to different species (36:4, 36:3, and 38:4, respectively). The peaks at *m/z* 873.6 and 899.6 can be assigned to the plasmenyl-PI with chains 38:3 and 40:4, in agreement with literature (Chen et al., 2016). Minor peaks attributable to other PI species are also listed in Table 3.

In the low *m/z* range of the mass spectrum of B2 corresponding to LPE, the main peak is at *m/z* 478.3 attributable to LPE containing an oleic acid chain (18:1). Furthermore, other minor peaks are present at *m/z* 452.3, 474.2, and 480.3 attributable to LPE 16:0, 18:3, and 18:0, respectively. The peak at *m/z* 494.3 is attributable to plasmenyl-LPE (20:0).

The mass spectrum of B3, acquired in negative ion mode, shows peaks attributable to PS species; the main peaks are at *m/z* 782.6 (36:4) and 784.6 (36:3), listed in Table 3 with other minor PS peaks.

B5, analyzed in negative ion mode, shows two groups of peaks attributable to PE and PG; the main peaks attributable to PE species are at *m/z* 738.5, 740.5, 742.5, and 766.5. In this band are also present the peaks at *m/z* 745.5 and 747.5, corresponding to PG.

In the mass spectrum of B6, acquired in negative ion mode, the peaks in the range *m/z* 1430–1490 were assigned to CL species having chains constituted by 72 total carbon atoms divided in two clusters of species with different levels of unsaturations (see Table 3).

**FIGURE 4:**
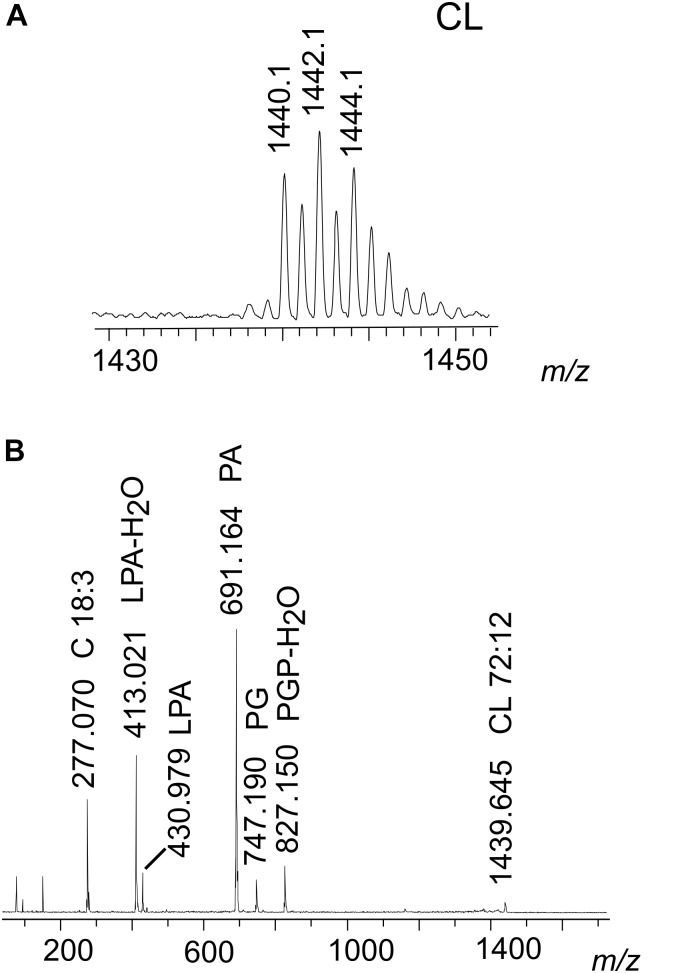
MALDI-TOF/MS analyses of the CL species of nauplii instar II. **(A)** Enlargement of range *m*/*z* 1430–1450 MALDI-TOF mass spectrum of CL band. Peaks at *m/z* 1440.1, 1442.1, and 1444.1 correspond to CL species containing four chains of C18 fatty acids with different number of double bonds. A detailed list of detected peaks of CL is shown in Table 3. **(B)** PSD analysis of the peak at *m/z* 1440.1 from CL band was performed. In the fragmentation patterns of CL, ion fragments correspond to: PGP-H_2_O (*m/z* 827.1), PG (*m/z* 747.1), PA (*m/z* 691.1), LPA (*m/z* 430.9), LPA-H_2_O (*m/z* 413.0), and the fatty acid 18:3 (*m/z* 277.0).

In Figure 4A we show the range *m/z* 1430–1450 of the MALDI mass spectrum corresponding to CL (band B6 of Figure 3B). To have information about the CL structures, PSD analyses have been performed. The PSD spectrum of CL at *m/z* 1440 is shown in Figure 4B; this analysis showed that the main CL species in the nauplii carries four linolenic acid chains, as previously reported in the literature (Chen et al., 2016).

### TLC of Neutral Lipids: Free Fatty Acids and DAGs Are More Abundant in the Nauplii Than in the Cysts

**FIGURE 5:**
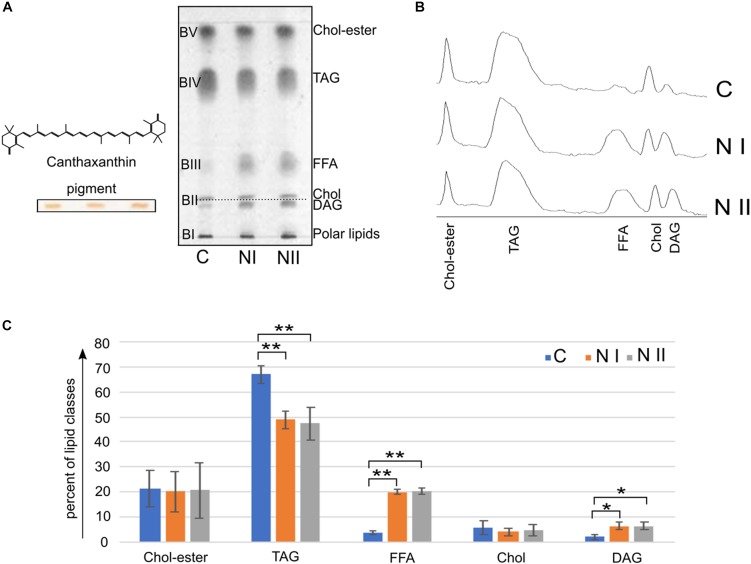
Neutral lipid classes in *A. franciscana*. **(A)** Shows TLC profiles of the neutral lipid extracts of cysts (C), nauplii instar I (NI), and nauplii instar II (NII); 80 μg of each total lipid extract was loaded in each lane. Lipids were detected by spraying with 5% sulfuric acid in water, followed by charring at 180°C for 5 min. BI corresponds to polar lipids; the lipid bands from BII to BV correspond to neutral lipids. BII is a double band, the lower band contains canthaxanthin (orange before staining, see band on the left of the plate) and diacylglycerols (DAG), the upper band was assigned to cholesterol (Chol); BIII, BIV, and BV were assigned to: free fatty acids (FFA), triacylglycerols (TAGs), and cholesteryl esters (Chol-esters), respectively. The molecular structure of the canthaxanthin pigment is shown over the band. **(B)** Shows chromatograms corresponding to the TLC lipid profiles in **(A)**. **(C)** Illustrates the relative densitometric intensity of Chol-esters, TAG, FFA, Chol, and DAG bands after averaging the chromatograms of three replicates of C, NI, and NII samples (as described in the Materials and Methods section). ^∗^*P* < 0.05, ^∗∗^*P* < 0.01 by Tukey’s *post hoc* test.

Although all packed at the solvent front in the upper region of TLC profiles shown in Figure 2A, it can be roughly seen that the neutral lipid components change going from cysts to nauplii. For this reason, we further analyzed the neutral lipids of C, NI, and NII by TLC with an eluent designed to highlight various neutral lipids in the total extracts (Figure 5A). After the chromatographic run, polar lipids remain at the bottom of TLC plate (BI), while a number of bands of neutral lipids were separated along the plate until the solvent front (BII – BV).

Neutral lipids were identified by their *R*_f_ values relative to standard markers (not shown).

In BII there are two bands or lipid species. The *R*_f_ value of the upper band in BII corresponds to that of cholesterol, while the lower band corresponds to the pigment canthaxanthin (naturally orange before staining, see TLC bands on the left of Figure 5A); no differences in the cholesterol and pigment bands can be seen in the different *Artemia* development stages. In addition based on migration of authentic standards (not shown), we could assess that diacylglycerol (DAG) co-migrates with canthaxanthin in BII. By comparing lower band in BII in the three stages, we can see that DAG is more abundant in the naupliar stages.

The spot in BIII, corresponding to the free fatty acids (FFA), is more abundant in the naupliar stages than in the cysts.

The BIV band contains triacylglycerols (TAGs); these neutral lipids are the most abundant component in all three lipid profiles; in addition, by comparing the three lanes, it can be seen that the cysts contain higher content of TAGs than naupliar stages.

Finally, BV is relative to cholesteryl -esters and no differences can be seen in the different samples.

Chromatograms shown in Figure 5B correspond to the three TLC neutral lipid profiles in Figure 5A.

By performing densitometric analyses of neutral lipid profiles and statistical analysis, we confirmed the significantly changes of TAG, DAG and FFA amounts during the development, from cysts to the first two naupliar stages.

The histogram in Figure 5C shows the relative amount of neutral lipids in the three-growing states: cysts contain 67% of TAG that decrease at 49–47% in NI and NII; while FFA and DAG levels are very low in the cysts (3.7 and 2%, respectively) and significantly increase at 20% (FFA) and 6% (DAG) in both naupliar stages. Cholesterol and Colesteryl-esters levels do not change much during the development.

Percent values of different neutral lipids and statistical results are reported in the Table 4.

**Table 4:** Neutral lipid classes identified in cysts, nauplii I, and nauplii II.

Neutral lipid classes	% total neutral lipid		

	Cysts	Nauplii I	Nauplii II
Chol-esters	21.3 ± 7.2	20.3 ± 8.1	20.7 ± 11.1
TAG	67.0 ± 3.5	49.0 ± 3.5	47.3 ± 6.6
FFA	3.7 ± 0.6	20.0 ± 1.0	20.3 ± 1.1
Chol	5.7 ± 2.9	4.0 ± 1.7	4.7 ± 2.1
DAG	2.0 ± 1.0	6.3 ± 1.5	6.3 ± 1.5


*Data are means ± SD of triplicates expressed in percent total neutral lipids. One-way ANOVA was performed [F_(2,__6)_= 15.07, p = 0.005 for TAG; F_(2,__6)_= 385.4, p = 4.61e-07 for FFA; F_(2,__6)_= 9.98 p = 0.01 for DAG] followed by post hoc Tukey’s test.*

The main species of DAG and TAG recognized by MALDI-TOF/MS analyses of the fraction of neutral lipids isolated after precipitation in cold acetone (as described in Materials and Methods) are reported in Table 2.

## Discussion

Lipids are the major source of metabolic energy and are involved in several cellular processes for growth, reproduction and survival of initial larval stages. Besides the structural role, phospholipids are important cofactors of membrane proteins and substrates of enzymes activated by the signal transduction cascades. Cholesterol, an important lipid component of cell membrane structure and hormone precursor, is an essential nutrient for crustaceans; it plays a fundamental role in endocrine functions such as for the synthesis of hormones, like ecdysteroids and sesquiterpenoids, regulating the development (Sánchez-Paz et al., 2006).

In *Artemia* very few molecular data on the role of lipids in physiological phenomena as well as on the enzymes involved in lipid metabolism are available in the literature. Information on changes of lipid composition during the development of *Artemia* is fragmentary or missing.

Some studies have been carried out in adults, whereas other analyses have been reported for nauplii, in most cases as complementary information to the main aim of the work. In some studies lipid analyses on cysts were performed without taking into account the presence in the sample of nauplii at the very initial state of development (Enzler et al., 1974; Gallagher and Brown, 1975; Webster and Lovell, 1990).

Variations in the lipid content and the fatty acid composition during the first 24 h of life of *Artemia* nauplii were studied using fresh-water-type samples from Great Salt Lake and San Francisco Bay populations (Claus et al., 1979). Soon after, Persoone et al. (1980) also reported data on variations in the lipid content and fatty acid composition of lipids from cysts to newly hatched nauplii from several strains. In the 1991, Navarro et al. (1991) analyzed the variations of lipid classes during the first development stages using as a model cysts and nauplii from parthenogenetic diploid strain of *Artemia* from the “La Mata” lagoon (Torrevieja, Alicante, Spain). The different lipid classes (lysophospholipids, phospholipids, pigments, cholesterol and other neutral lipids) were identified by high performance TLC. Navarro et al. showed a decrease in the percentage of fatty acids 16:0 and 16:1n-7 and an increase in 20:5n-3 from cysts to naupliar stages and a decrease in the PC/PE ratio. The implications of these findings for the use of *Artemia* sp. as a larval feed in aquaculture were widely discussed (Navarro et al., 1991).

The only studies in the literature on lipids of *Artemia* based on MALDI-TOF/MS analysis deal with isolated sphingoids, sphingolipids and glycosphingolipids. Kojima et al. studied in details some complex lipids of cysts of *A. franciscana*. In particular they characterized minor sphingolipids and glycosphingolipids isolated and purified from the total lipid extract; they discovered that cysts contain novel structures of glycosphingolipids and fucosylglycosphingolipids not found in other animal species (Kojima et al., 2010, 2011, 2013).

We could not detect such complex glycosphingolipids in our study, however, other sphingoid assignments well correspond to those described in the above studies.

In the present work we used for the first time combined MALDI-TOF/MS and TLC analyses to compare the main lipid classes during the development from cysts to two naupliar stages, in order to possibly gain preliminary insights into lipid metabolism of *Artemia*.

In general, our data on cysts are in good agreement with those of Chen et al. (2016) and, in addition, they neatly document basic changes in the lipid species during the first stages of development.

Chen et al. assembled the total and mitochondrial lipid profile of *A. franciscana* cysts by classic shot gun lipidomics (Chen et al., 2016). The number of various lipid species and their abundance in cysts has been assessed in the total lipid extract and the extract of isolated mitochondria. TAGs were the most abundant components in the range from zero to 100 mg of lipid/mg protein followed by PC species; LPE, LPC, and SM were quantified in the order of 1–2 mg lipid/mg proteins and finally LPA, LPG, and LPI plus other minor components were found to be present at levels lower than 0.1 mg lipid/mg proteins. In the study of Chen et al. (2016) particular attention has been dedicated to CL as lipid marker of mitochondria. As expected, due to the presence of four positions for lipid chains in the molecule, a big number of CL species have been identified in mitochondria and mitoplasts of cysts; furthermore a number of monolysocardiolipin and dilysocardiolipin species were also described (Chen et al., 2016).

Although with some limitations, we show that many aspects of the lipid composition of *Artemia* can be elucidated by our method of analyses. Data on lipid species, chains, polar and neutral lipids have been obtained. In our study neutral lipids, PC, SM, and LPC have been analyzed by MALDI-TOF/MS in the positive ion mode; in parallel CL, PE, PG, PS, PI, LPE have been analyzed in the negative ion mode of analysis.

Lipid extracts of *Artemia* show a preponderant amount of neutral lipids compared to polar lipids.

The abundance of neutral lipids in cysts (80% of total lipids Navarro et al., 1992) and nauplii (60%, present paper) indicates a great availability of energy as storage lipids in these forms of life, but it could also suggest an important physiological role of these lipids in determining the buoyancy of planktonic crustaceans. Here we show for the first time that DAG, the precursor of phospholipid synthesis, also increases (at the expense of TAG) during the development. DAG represents the backbone for the formation of PC within the Kennedy pathway, whose enzymes were studied in the past in microsomal preparations from nauplii of the brine shrimp *Artemia salina* (Ewing and Finamore, 1970). DAG not only plays a role in the lipid biosynthetic reactions, but it is also an important lipid second messenger in signal transduction.

As regards polar lipids, our data indicate that the ratio PC/PE decreased progressively from cysts to nauplii, in agreement with above observations of Navarro et al.

For the first time we document the raise of CL and lysophospholipids levels, indicating an increase in complexity of metabolic pathways from cysts to naupliar stages. These changes are clearly associated with differentiation, with the increase of the number of molecular functions, as well as with the development of the organs and their specialization in nauplii.

The increase of CL in larvae well matches the formation of new cristae during the development, which requires the neosynthesis of the proteins of the respiratory complexes and of the accompanying phospholipids. It clearly suggests a close relationship between the CL content and the maturation of mitochondria from cysts to nauplii, and further sheds light on the important role of CL in the mitochondrial functions. Interestingly two different clusters of CL peaks have been found in the CL band isolated by TLC that could have different physiological roles in nauplii.

Lysophospholipids are bioactive compounds able to interact with protein membrane receptors and channels, such as the G-protein coupled receptors (GPCRs) and the transient receptor potential channels (TRPs) operating as cellular sensors and involved in various sensory pathways activated by light, chemical and temperature stimuli (Rivera and Chun, 2008; Zheng, 2013).

Finally, many plasmalogens have been identified in the phospholipid bands isolated by preparative chromatography, some species of PC and LPC, LPE plus many species of PI. Plasmenyl phospholipids are known to release cell-signaling molecules, affecting intracellular signaling cascades.

## Conclusion

In conclusion, our study has generated general information on major and minor polar lipid components of cell membranes together with neutral lipids in different forms of life of *Artemia*. Following our expectations, we have documented an increase of levels of bioactive lipids in naupliar stages in correspondence to the increasing complexity of the form of life. The data here presented may represent the basis for future studies in order to understand how environmental factors such as salinity, temperature and pH affect the lipid composition of cell membrane associated with significant effects on the physiology of the whole organism and its development.

## Author Contributions

AC and GVS designed the research. PL and RL-d-S performed the research. AC, PL, and SL analyzed the data and wrote the manuscript.

## Conflict of Interest Statement

The authors declare that the research was conducted in the absence of any commercial or financial relationships that could be construed as a potential conflict of interest.

## Footnotes

1 http://www.lipidmaps.org

## Abbreviations

9-AA: 9-aminoacridine
C: cysts
CL: cardiolipin
Chol: cholesterol
Chol-esters: cholesteryl-esters
DAG: diacylglycerol
FFA: free fatty acids
LPA: lysophosphatidic acid
LPC: lysophosphatidylcholine
LPE: lysophosphatidylethanolamine
MALDI-TOF/MS: matrix assisted laser desorption ionization time of flight mass spectrometry
NI: nauplii instar I
NII: nauplii instar II
PA: phosphatidic acid
PC: phosphatidylcholine
PE: phosphatidylethanolamine
PG: phosphatidylglycerol
PGP: phosphatidylglycerophosphate
PI: phosphatidylinositol
PS: phosphatidylserine
PSD: post-source decay
SM: sphingomyelin
TAG: triacylglycerol
TLC: thin layer chromatography
